# Electrical stimulation for limb spasticity in children with traumatic brain injury: Study protocol for a systematic review of randomized controlled trial

**DOI:** 10.1097/MD.0000000000014515

**Published:** 2019-02-22

**Authors:** He Wang, Guang-fu Song, Jing Nie, Xiao-hao Xu, Ying Zhang, Jin-rui Liu

**Affiliations:** a Department of Neurosurgery, First Affiliated Hospital of Jiamusi University; b First Ward of Pediatrics Department, First Affiliated Hospital of Jiamusi University; c Third Ward of Neurology Department, Jiamusi Central Hospital, Jiamusi; d Department of Neurology, Heilongjiang Electricity Hospital, Harbin, China.

**Keywords:** effect, electrical stimulation, limb spasticity, safety, systematic review, traumatic brain injury

## Abstract

**Background:**

Previous clinical studies have reported that electrical stimulation (ES) can be utilized to treat children with limbs spasticity (LS) after traumatic brain injury (TBI). Currently, no systematic review has addressed the effect of ES in children with LS following TBI. Thus, this systematic review will assess the effect and safety of ES for the children with LS after TBI.

**Methods:**

We will conduct the present systematic review of randomized controlled trials that will be retrieved from searches of PubMed, PsycINFO, WOS, Scopus, OpenGrey, Google Scholar, Cochrane Central Register of Controlled Trials, Embase, Cumulative Index to Nursing and Allied Health Literature, Allied and Complementary Medicine Database, and Chinese Biomedical Literature Database from the inception to the date of the literature searched. In addition, the clinical register websites, and reference lists of relevant studies will also be searched. Two independent reviewers will evaluate the eligibility criteria for all papers, extract the data and determine the methodology quality by using Cochrane risk of bias tool.

**Results:**

The results of this systematic review will pool the latest available data, and are expected to provide the summary of present evidence of ES for children with LS following TBI.

**Timeline:**

This systematic review will start on January 10, 2019 and expected to complete by June 1, 2019.

**Ethics and dissemination:**

No research ethic approval is needed in this study, because the data of this systematic review will not base on the individual data level. The results will be disseminated to publish at peer-reviewed journals or will present at relevant conferences.

**PROSPERO registration number:**

CRD42019120037

## 1 Introduction

Traumatic brain injury (TBI) is one of the leading causes of death and disability,^[1,2]^ especially among the population of children, teens, and the elderly.^[3–5]^ This disorder often results in chronic neurological, cognitive, and behavioral impairments, psychological conditions, and limbs paralyzed or spasticity.^[6–14]^ Thus, if this condition cannot be treated timely and effectively, it may lead to very poor quality of life for patients who experience such disorder.^[15,16]^

Previous clinical studies have reported that electrical stimulation (ES) can be used to treat TBI, and its complications effectively.^[17–19]^ However, no systematic review has explored the effect and safety of ES for the treatment of children with limbs spasticity (LS) after traumatic brain injury (TBI). Thus, this systematic review will aim to assess the effect and safety of ES for the treatment of LS following TBI among children population.

## 2 Methods and analysis

### 2.1 Study registration

This study has been registered at PROSPERO with number of CRD42019120037. This protocol is reported based on the Preferred Reporting Items for Systematic Reviews and Meta-Analysis Protocol statement guidelines.^[20]^

### 2.2 Inclusion criteria for study selection

#### 2.2.1 Type of studies

This systematic review will only include randomized controlled trials (RCTs) of ES for the treatment of children with LS following TBI. The other studies including non-RCTs, quasi-RCTs, reviews, comments, letters, case reports, case series, and animal studies will all not be considered.

#### 2.2.2 Type of participants

All patients under 18 years old with LS after TBI will be included. However, patients will be excluded if they had LS before the TBI or the LS resulted from other disorders, such as stroke, cancers, spinal cord injury, and any other conditions, except TBI.

#### 2.2.3 Type of interventions

This systematic review will include any forms of ES, including neuromuscular electrical stimulation, transcutaneous electrical nerve stimulation, electrical muscle stimulation, Russian electrical stimulation, functional electrical stimulation, as well as the electroacupuncture. However, the combination therapy of ES with any other interventions will not be considered. As for control therapy, any kinds of interventions are allowed, except any forms of ES.

#### 2.2.4 Type of outcomes

Primary outcome is limb spasticity status, as assess by the Modified Ashworth Scale, or any other related scales. The secondary outcomes include limb function, as measured by Disability Assessment Scale or other related scales; and health-related quality of life, as evaluated by Assessment of Quality of Life, or other associated scales. In addition, adverse events will also be assessed.

#### 2.2.5 Search strategy

We will retrieve the following bibliographic databases and relevant sources for all potential eligible trials: PubMed, PsycINFO, WOS, Scopus, OpenGrey, Google Scholar, Cochrane Central Register of Controlled Trials, Embase, Cumulative Index to Nursing and Allied Health Literature, Allied and Complementary Medicine Database, and Chinese Biomedical Literature Database, as well as the clinical register websites, and reference lists of relevant studies. All these sources will be searched from the inception to the date of studies retrieved. The search strategy details for Cochrane Central Register of Controlled Trials are presented in Table 1. Equivalent search strategies will be applied to other databases. Moreover, it will also be translated into Chinese and then will be applied to Chinese databases. All databases and other sources will be searched without language restrictions.

**Table 1:**
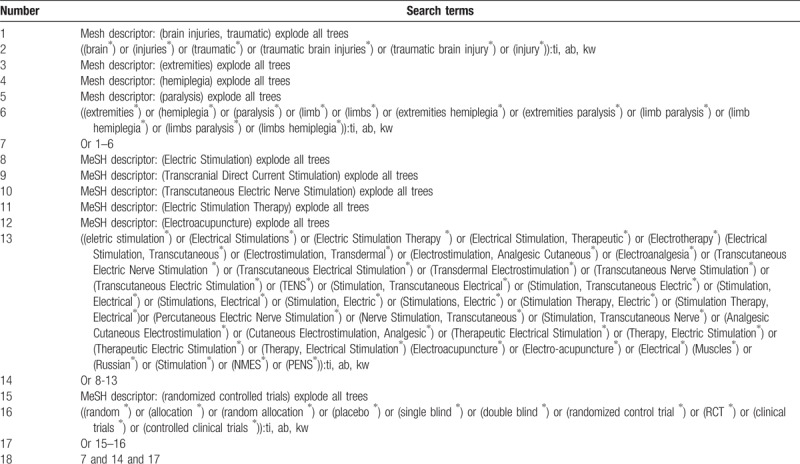
Search strategy applied in CENTRAL database.

### 2.3 Data collection and management

#### 2.3.1 Study selection

**Figure 1:**
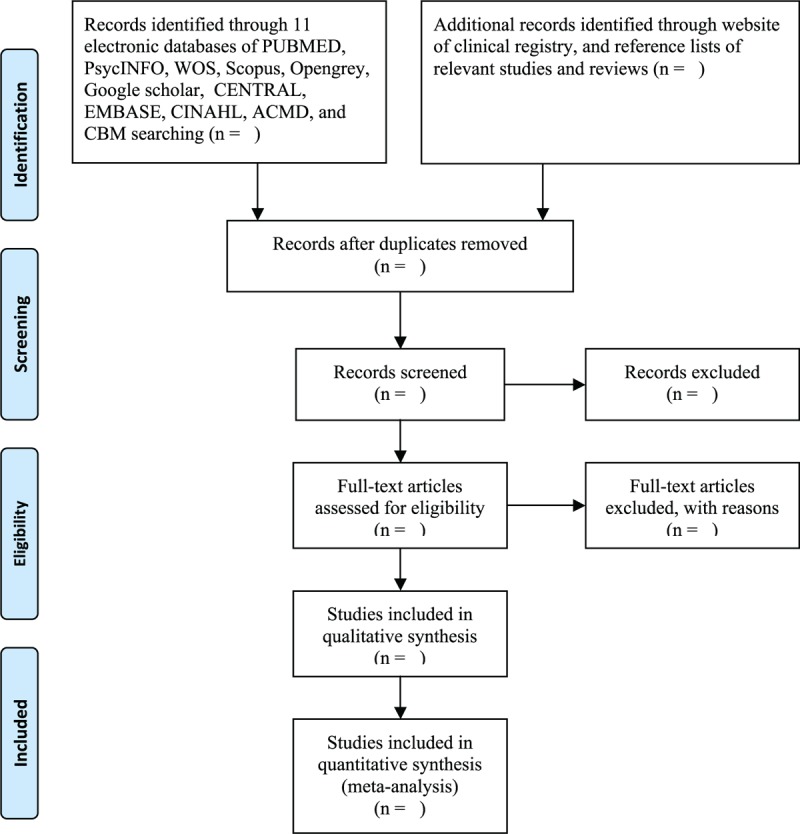
Process of study selection.

The software of EndNote X8 will be utilized to manage the records of electronic databases. The first stage of the initial screen will involve scanning titles and abstracts according to the previous defined eligibility criteria. After the first stage, full texts will be reviewed by reading the full papers to further assess them if they meet all eligibility criteria. All selection procedures will be conducted by 2 independent review authors. Any divergences regarding the study selection between them will be tackled by a third review author through discussion. The process of study selection is presented in Figure 1.

#### 2.3.2 Data extraction and management

The form of data extraction has been built prior to the data collection. The following data information will be extracted by 2 independent review authors: general information (title, first author, publication year, and location), study methods (study design, sample size, details of randomization, concealment, blinding, insufficient reports information, as well as other sources of bias), participants (age and sex information, inclusion and exclusion criteria), interventions of both experimental and control groups (types of interventions, duration, frequency, intensity, and dosage), and outcomes (primary, secondary, and any other outcome information). All disagreements regarding the data collection will be handled by consensus with a third review author.

#### 2.3.3 Missing data dealing with

Any types of missing data will be required by contacting the primary authors. If these data can be provided after the requirement, the comprehensive data will be pooled, and meta-analysis will be conducted. If these data cannot be provided, then the available data will be still pooled, and meta-analysis will be performed with limitation discussion of the missing data.

#### 2.3.4 Methodology quality assessment

The methodology quality will be assessed by 2 independent review authors through the Cochrane risk of bias tool. All judgments will be fully described, and conclusions will be presented with low risk of bias, unclear risk of bias, or high risk of bias. Any disagreements will be solved by consensus or discussion with a third review author.

### 2.4 Data synthesis and analysis

#### 2.4.1 Measurement of treatment effect

RevMan 5.3 software and STATA 12.0 software will be utilized to pool the data and to conduct the meta-analysis. As for continuous data, the pooled results will be presented as mean difference and 95% confidence intervals (CIs). As for dichotomous data, the pooled results will be summarized as risk ratio and 95% CIs. Value of *P < *.05 is considered as having statistical significance.

#### 2.4.2 Assessment of heterogeneity evaluation and results data pooled

The Cochrane *Q* statistic and *I*^*2*^ index will be used to identify heterogeneity across the included studies. We will consider the significant heterogeneity if *I*^*2*^ >50% and/or *Q* test<0.10, and random-effect model will be used to pool the results data. Otherwise, fixed-effect model will be utilized to pool the results data.

#### 2.4.3 Subgroup analysis

If the heterogeneity is significant, then subgroup analysis will be performed to detect the feasible factors that may cause the heterogeneity. It will be performed according to the different types of treatment, controls, durations, and outcomes.

#### 2.4.4 Sensitivity analysis

The sensitivity analysis will be conducted to check the robustness and stability of pooled data, methodological quality, and the missing data.

#### 2.4.5 Publication bias

When sufficient eligible studies are included (at least 10 trials), the funnel plot will be conducted to detect that if there exists potential publication bias.^[21]^ Meanwhile, Egger linear regression test will also be conducted to check the funnel plot asymmetry.^[22]^

## 3 Discussion

This protocol of systematic review will summarize the up-to-data outcome data to assess the effect and safety of ES in children with LS after TBI. The findings of this study will provide the evidence whether ES will achieve promising benefits in children with LS following TBI. Nevertheless, the safety of ES treatment will also be assessed. Moreover, the strength of the findings will be summarized by the assessment of Cochrane risk of bias tool. Findings of this systematic review and meta-analysis will provide helpful evidence for the clinicians to make decisions in the clinical practice, as well as for the health policy maker.

## Author contributions

**Conceptualization:** He Wang, Guang-fu Song, Jing Nie, Xiao-hao Xu, Jin-rui Liu.

**Data curation:** He Wang, Guang-fu Song, Jing Nie, Xiao-hao Xu, Ying Zhang.

**Formal analysis:** He Wang, Guang-fu Song, Jing Nie.

**Investigation:** Jin-rui Liu.

**Methodology:** He Wang, Guang-fu Song, Jing Nie, Xiao-hao Xu, Ying Zhang.

**Project administration:** Jin-rui Liu.

**Resources:** He Wang, Guang-fu Song, Jing Nie, Xiao-hao Xu, Ying Zhang, Jin-rui Liu.

**Software:** He Wang, Guang-fu Song, Xiao-hao Xu.

**Supervision:** Jin-rui Liu.

**Validation:** Guang-fu Song, Ying Zhang, Jin-rui Liu.

**Visualization:** Guang-fu Song, Ying Zhang, Jin-rui Liu.

**Writing – original draft:** He Wang, Jing Nie, Xiao-hao Xu, Ying Zhang.

**Writing – review & editing:** He Wang, Guang-fu Song, Jing Nie, Xiao-hao Xu, Ying Zhang, Jin-rui Liu.
